# PTPN2 negatively regulates macrophage inflammation in atherosclerosis

**DOI:** 10.18632/aging.202326

**Published:** 2020-12-19

**Authors:** Xiaorong Hu, Ruisong Ma, Jianlei Cao, Xianjin Du, Xinyong Cai, Yongzhen Fan

**Affiliations:** 1 Department of Cardiology, Zhongnan Hospital of Wuhan University, Wuhan, Hubei, China; 2 Department of Cardiology, The First College of Clinical Medical Sciences, China Three Gorges University, Yichang, Hubei, China; 3 Department of Emergency, Renmin Hospital of Wuhan University, Wuhan, Hubei, China; 4 Department of Cardiology, Jiangxi Provincial People's Hospital Affiliated to Nanchang University, Nanchang, JiangXi, China

**Keywords:** PTPN2, inflammation, macrophage, atherosclerosis

## Abstract

Atherosclerosis is the main cause of cardiovascular disease. Systemic inflammation is one important characteristic in atherosclerosis. Pro-inflammatory macrophages can secrete inflammatory factors and promote the inflammation of atherosclerosis. It has a great value for the treatment of atherosclerosis by inhibiting the release of inflammatory factors in macrophages. However, the detailed mechanism of this process is still unclear. In this study, we constructed an APOE^-/-^ mice model of atherosclerosis to research the molecular mechanism of atherosclerosis. Protein tyrosine phosphatase non-receptor type 2 (*PTPN2*), an anti-inflammatory gene, was dramatically decreased in inflammatory mice. Deletion of *PTPN2* could significantly induce monocytes toward M1 phenotype of macrophages, enhance the secretion of IL-12 and IL-1, and promote cell proliferation, invasion and metastasis. Mechanism research showed that *PTPN2*-mediated p65/p38/STAT3 de-phosphorylation could block the process of macrophage inflammation. In vivo experiments showed that *PTPN2* may effectively inhibit the inflammatory response during atherosclerosis. In conclusion, we uncovered the negative role of *PTPN2* in the occurrence of atherosclerosis, and this study provides a new potential target for atherosclerosis treatment.

## INTRODUCTION

Cardiovascular disease is the leading cause of mortality in the world [1]. Atherosclerosis is the main cause of cardiovascular disease, the dominant global health threat, and a huge burden for society [2, 3]. Atherosclerotic plaque and symptoms are two typical characteristics of atherosclerosis in early stage [4]. The plaques will become larger and more unstable with the progress of atherosclerosis. This leads to the obstruction of blood flow, and induces myocardial infarction or stroke [5]. Atherosclerosis is also a chronic inflammatory disease [6]. Accumulation of immune cells and necrotic debris, and endothelial dysfunction are its typical characteristics. This will lead to vascular endothelium formation of atherosclerotic plaques [7–9]. Systemic inflammation is another feature of this disease, and high levels of C-reactive protein (CRP) is a biomarker for it [10, 11]. Despite decades of research, the molecular mechanism underlying atherosclerosis is still unclear.

Persistent systemic inflammation is one typical characteristic in atherosclerosis. Exploring its molecular mechanisms has a great value for atherosclerosis treatment. Studies have found that a large number of immune cells are present in atherosclerotic lesions [12]. The main cells among these are macrophages [13]. Macrophages are mainly derived from monocytes which differentiation in the intima of arteries [14]. This process is mainly affected by the local microenvironment [15], the metabolic status of macrophages [16], and some epigenetic factors [17]. But the detailed molecular mechanism is unclear. The main types of macrophages include M1 and M2 types, which are also called as pro-inflammatory and anti-inflammatory macrophages, respectively [18, 19]. M1 macrophage could secrete multiple pro-inflammatory factors, such as TNF-α, IL-1α, IL-1β, IL-12, as well as the chemokine CXCL9, CXCL10, ROS and nitric oxide (NO) [20–22]. These inflammatory cytokines, chemokines, and ROS may cause the continuous inflammation around atherosclerotic plaques, the recruitment of inflammatory cells, and the formation of plaque [23, 24]. All of these researches illuminate the important role of macrophages in the process of atherosclerosis. However, the detailed regulation mechanism of inflammatory factors secreted by macrophages remains unclear. It is still lacking effective target for atherosclerosis treatment.

In this study, we performed a preliminary study on the molecular mechanism of atherosclerosis, and attempted to find an effective target to block the occurrence of macrophage inflammation and atherosclerotic plaque. We constructed an APOE^-/-^ mice model of atherosclerosis, and found that *PTPN2* was dramatically decreased in inflammatory mice. Loss-of-function study showed that, *PTPN2* deficiency could induce monocytes to M1 phenotype of macrophages, enhance the secretion of IL-12 and IL-1β, and promote cell proliferation, invasion and metastasis. Mechanism research indicated that *PTPN2*-mediated p65/p38/STAT3 de-phosphorylation could block macrophages inflammation. In vivo experiments showed that *PTPN2* could inhibit the inflammatory response in atherosclerosis. We uncovered the negative role of *PTPN2* in macrophages inflammation, and this work may provide a new potential target for atherosclerosis treatment.

## RESULTS

### *PTPN2* is negatively correlated with inflammation in ApoE^-/-^ mice

**Figure 1:**
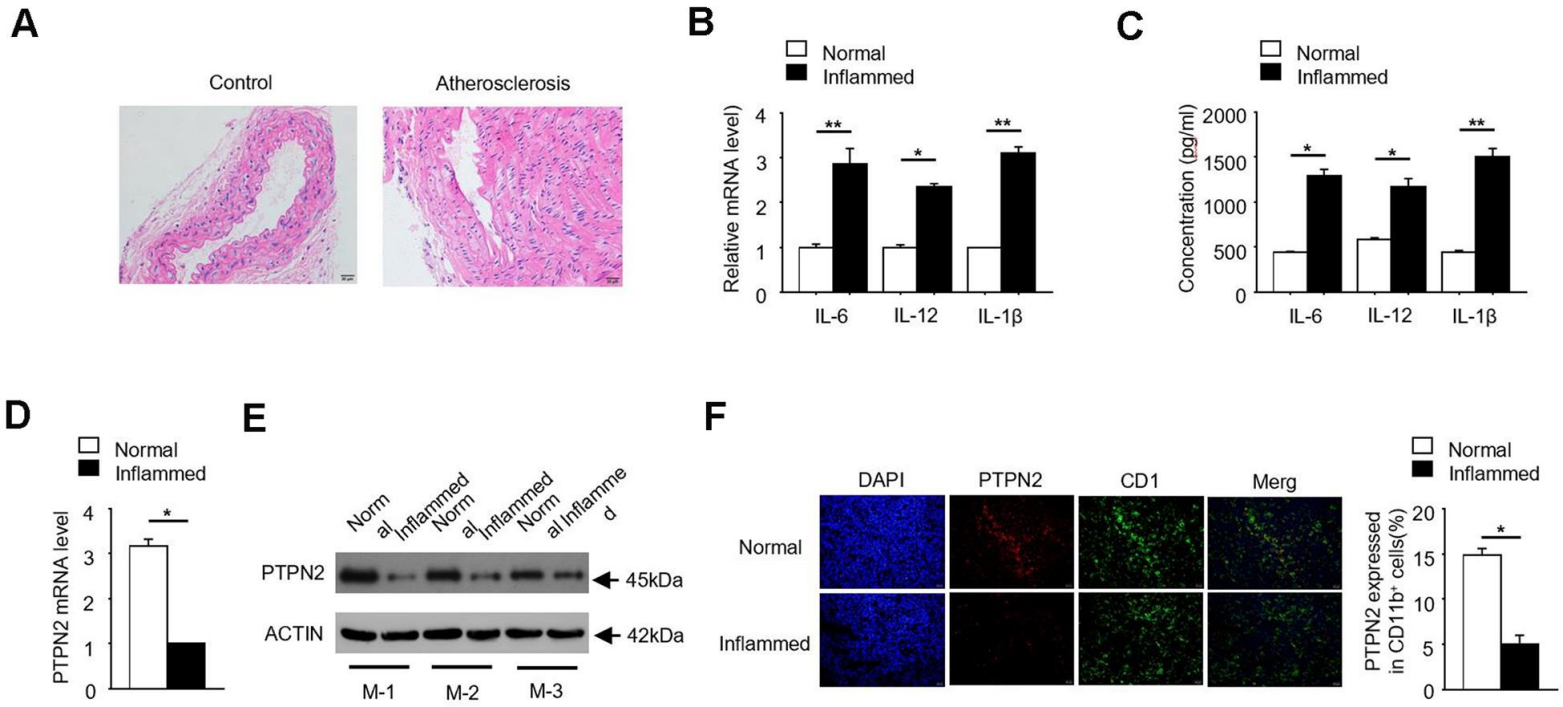
***PTPN2* is negatively correlated with inflammation in ApoE^-/-^ mice.** (**A**) Photomicrograph of atherosclerotic lesions stained with Hematoxylin-Eosin. (**B**) The mRNA level of *IL-6*, *IL-12* and *IL-1β* in macrophages from normal and inflamed aortic roots were analyzed by qRT-PCR. (**C**) ELISA assay analysis the production of IL-6, IL-12, IL-1β in macrophages from normal and inflamed fictions. (**D**) *PTPN2* mRNA level in normal and inflamed aortic roots were analyzed by qRT-PCR. (**E**) IB analysis PTPN2 expression from three different ApoE^-/-^ mice. M-1, M-2, M-3 were three different ApoE^-/-^ mice. (**F**) Confocal microscopy analysis CD11b and PTPN2 in normal and inflamed aortic roots in ApoE^-/-^ mice. Data is representative of at least three independent experiments and are presented as mean ± SD. ns, not statistically significant; *, P < 0.05. **, P < 0.01.

In order to investigate the pathogenesis of atherosclerosis, we constructed an APOE^-/-^ mice model of atherosclerosis. The photomicrograph of atherosclerotic lesions stained with Hematoxylin-Eosin was showed in Figure 1A. Due to the release of inflammatory factors is one of its typical characteristics, we detected the expression of IL-6, IL-12 and IL-1β in normal and inflammatory aortic roots of ApoE^-/-^ mice. qRT-PCR assay showed that the mRNA levels of inflammatory factors IL-6, IL-12 and IL-1β were significantly increased in inflammatory APOE^-/-^ mice (Figure 1B). Consistently, the protein concentrations of IL-6, IL-12 and IL-1β were also significantly increased in inflammatory APOE^-/-^ mice (Figure 1C). PTPN2, which is one of non-receptor protein tyrosine phosphatases, is related to the occurrence of various inflammatory-linked diseases [25, 26]. In order to explore the relationship between *PTPN2* and inflammation in APOE^-/-^ mice, we detected the expression of *PTPN2* in normal and inflammatory APOE^-/-^ mice. qRT-PCR assay showed that the mRNA expression of *PTPN2* was significantly reduced in inflammatory APOE^-/-^ mice (Figure 1D). Consistently, the protein level of PTPN2 in three different inflammatory APOE^-/-^ mice was also lower than that in normal control mice (Figure 1E). In addition, we also analyzed the protein level of PTPN2 in normal and inflammatory APOE^-/-^ mice by confocal microscopy. Compared with the normal control, PTPN2 in inflammatory APOE^-/-^ mice was significantly reduced, while the expression level of CD11b has no obvious change (Figure 1F). These results indicate that the APOE^-/-^ mice model of atherosclerosis has obvious inflammation, and *PTPN2* is negatively correlated with inflammation in ApoE^-/-^ mice.

### *PTPN2* deficiency enhances the secretion of inflammatory cytokines in THP-1 cells and U937 cells, and toward M1 phenotype

**Figure 2:**
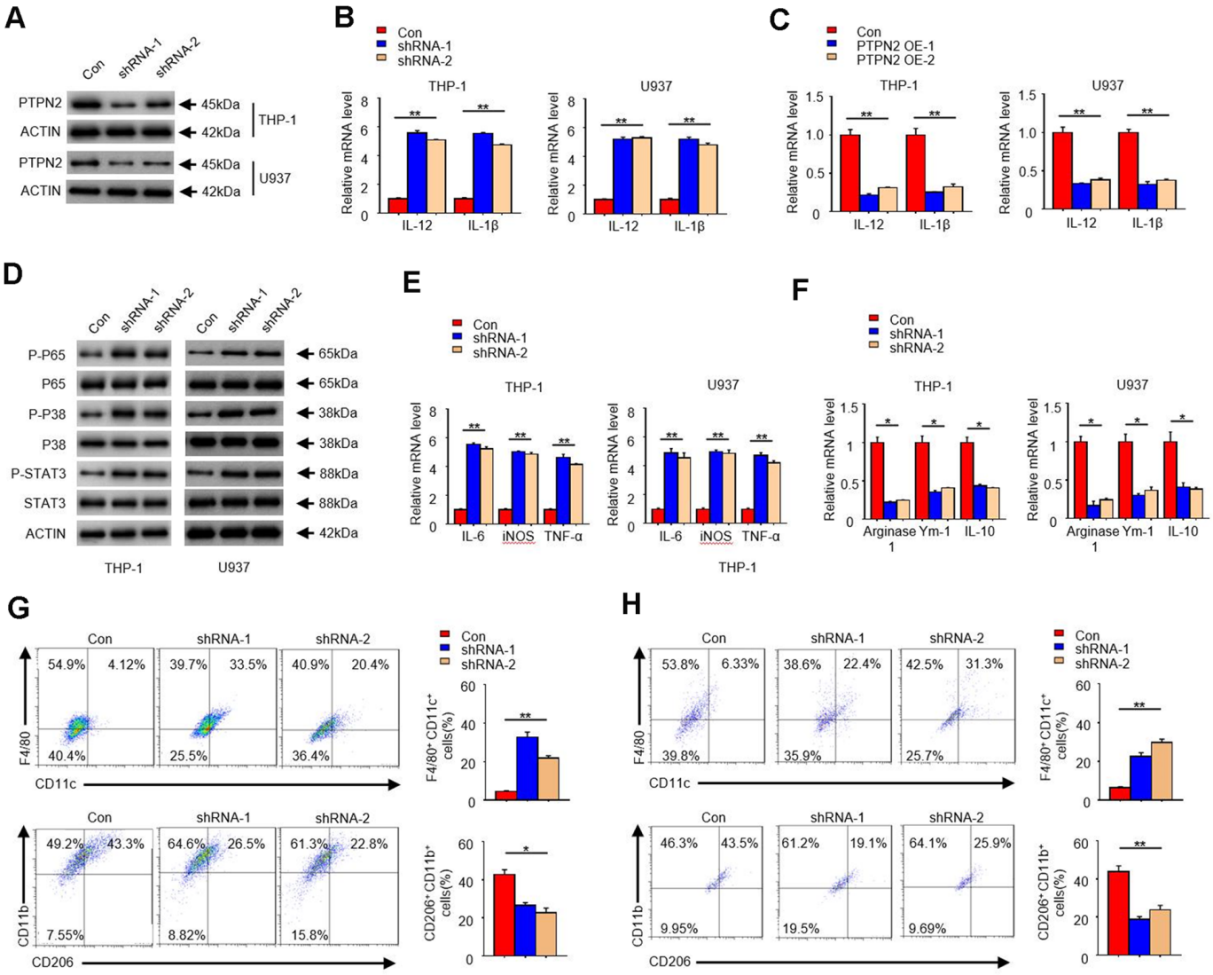
***PTPN2* deficiency enhances the secretion of inflammatory cytokines in THP-1 cells and U937 cells, and toward M1 phenotype.** shRNA-1 and shRNA-2 of *PTPN2* were used to knock down the expression of *PTPN2* in THP-1 cells and U937 cells. (**A**) IB analysis PTPN2 expression in THP-1 cells and U937 cells. (**B**) The mRNA level of *IL-12* and *IL-1β* in THP-1 cells and U937 cells were analyzed by qRT-PCR. (**C**) Expression of IL-12 and IL-1β in THP-1 cells or U937 cells with PTPN2 overexpression were detected by qRT-PCR. (**D**) IB analysis the protein levels of p65, p38 and STAT3 in THP-1 cells and U937 cells. (**E**, **F**) M1 and M2 related polarization genes were analyzed by qRT-PCR assay in THP-1 cells and U937 cells. (**G**, **H**) Flow cytometry analysis the polarization of THP-1 cells and U937 cells. Data are representative of at least three independent experiments and are presented as mean ± SD. ns, not statistically significant; *, P < 0.05. **, P < 0.01.

In order to reveal the function of *PTPN2* in the occurrence of atherosclerosis, we artificially synthesized two shRNA of *PTPN2* gene, and used the THP-1 cell and U937 cell as the model to research. Firstly, we transfected these two shRNA segments into THP-1 cell and U937 cell, and analyzed the protein of PTPN2 by Western blot assay. Results showed that both shRNA could significantly inhibit the expression of PTPN2 protein (Figure 2A). To explore the effect of *PTPN2* deficiency on the secretion of inflammatory factors, we examined the changes of IL-12 and IL-1β in THP-1cells and U937 cells. qRT-PCR assay showed that *PTPN2* deficiency could enhance the mRNA expression of *IL-12* and *IL-1β* (Figure 2B). Conversely, overexpression of *PTPN2* could also significantly inhibit the expression of IL-12 and IL-1β in THP-1 cells and U937 cells (Figure 2C). In addition, the activation of NF-κB signal pathway, MAPK signal pathway and JAK-STAT signal pathway are closely related to the secretion of inflammatory factors [27–29]. We also tested the phosphorylation levels of p65, p38 and STAT3 in THP-1 cells and U937 cells. Western blot experiments showed that shRNA-1 or shRNA-2 of *PTPN2* could increase the phosphorylation levels of p65, p38 and STAT3, but it has no obvious change on total p65, p38 and STAT3 proteins (Figure 2D). These results indicate that *PTPN2* deficiency induces the secretion of inflammatory factors by activating the p65/p38 /STAT3 signal pathway.

In addition, we also analyzed the polarization characteristics of THP-1 cells and U937 cells. As shown in Figure 2E, the expression of *IL-6*, *TNF-a*, and *iNOS*, which associated with M1 polarization, were significantly increased in *PTPN2* deficiency cells. Conversely, the expression of *Arginase1*, *Ym1*, and *IL-10*, which associated with M2 polarization, were reduced in *PTPN2* deficiency cells (Figure 2F). F4/80^+^ CD11C^+^ is the surface antigen marker of M1 phenotype, while CD206^+^ / CD11B^+^ is the surface antigen marker of M2 phenotype. Flow cytometry assays showed that THP-1 cell with F4/80^+^CD11C^+^ were significantly increased, and THP-1 cell with CD206^+^ / CD11B^+^ were significantly decreased after *PTPN2* was suppressed by its shRNA (Figure 2G). The results observed in U937 cells were consistent with those results in THP-1 cells (Figure 2H). All of these results showed that the loss of *PTPN2* will lead to enhance the secretion of inflammatory cytokines and toward M1 phenotype.

### *PTPN2* deficiency promotes the proliferation, migration and invasion of THP-1 cells and U937 cells

**Figure 3:**
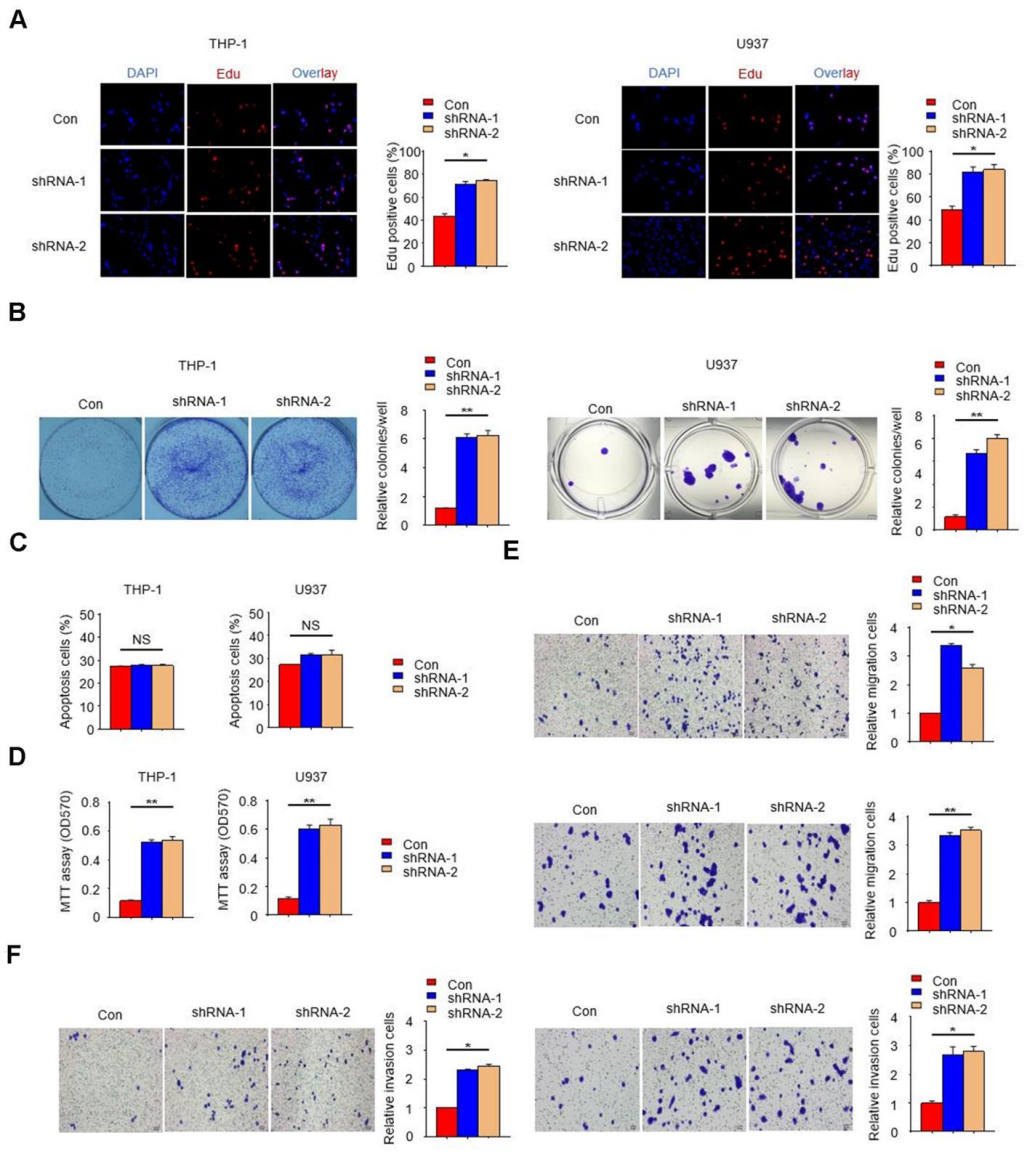
***PTPN2* deficiency promotes the proliferation, migration and invasion of THP-1 cells and U937 cells.** (**A**, **B**) EDU assay and colony formation assay were used to analyze the proliferation ability of THP-1 cells and U937 cells. (**C**) Apoptosis of THP-1 cells and U937 cells were evaluated by flow cytometry. (**D**) MTT assay were used to examine the viability of HUVEC cell after incubated with THP-1 cells or U937 cells. (**E**, **F**) Transwell migration and invasion assays were performed in THP-1 cells and U937 cells. The upper is THP-1 cells and the lower is U937 cells in Figure 3E. The left is THP-1 cells and the right is U937 cells in Figure 3F. Bar= 20μM. Data are representative of at least three independent experiments and are presented as mean ± SD. ns, not statistically significant; *, P < 0.05. **, P < 0.01.

In order to further reveal the effect of *PTPN2* deletion on THP-1 cells and U937 cells, we examined the cell proliferation ability with *PTPN2* deficiency by EDU assay. Results showed that the proliferation ability of THP-1 cells and U937 cells with *PTPN2* deficiency was enhanced when compared to control cells (Figure 3A). The proliferation and apoptotic capacity were also detected by clone formation assay and flow cytometry. Results showed that cells with *PTPN2* deficiency has stronger proliferation ability than the control cells (Figure 3B). Flow cytometry assay showed that there was no difference in the proportion of apoptotic cells between *PTPN2* deficiency cells and control cells (Figure 3C). In addition, we also incubated HUVEC cells with THP-1 cells or U937 cells for a period of time, and found that the proliferation ability of HUVEC cells was increased significantly (Figure 3D). These results showed that interference with the expression of *PTPN2* gene could promote cell proliferation, with no effect on its apoptosis. In addition, we also evaluated the migration and invasion ability of THP-1 cells and U937 cells. Results showed that deletion of *PTPN2* could enhance the migration and invasion of cells (Figure 3E, 3F). The above results suggested that *PTPN2* deficiency promotes proliferation, metastasis and invasion of THP-1 cells and U937 cells.

### *PTPN2* inhibitor XIX mimics the effects of *PTPN2* deletion in THP-1 cells

**Figure 4:**
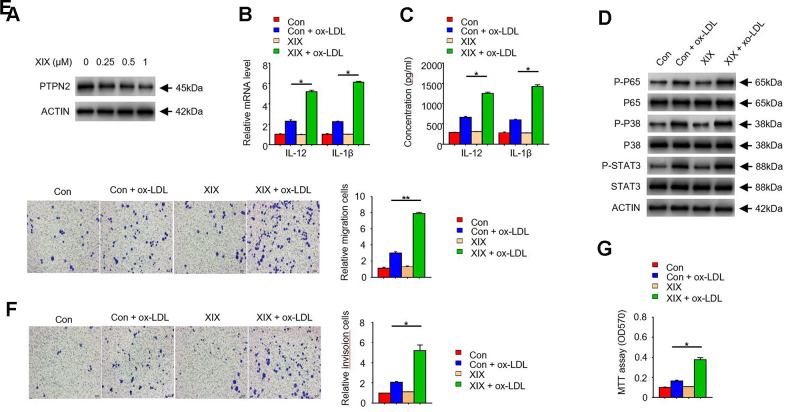
***PTPN2* inhibitor XIX mimics the effects of *PTPN2* deletion in THP-1 cells.** (**A**) Expression of *PTPN2* in THP-1 cells with different concentration of XIX were detected by IB assay. (**B**) mRNA levels of IL-12 and IL-1β in THP-1 cells were analyzed by qRT-PCR assay. (**C**) ELISA assay was used to analyze the production of IL-12 and IL-1β in THP-1 cells in the presence of XIX or ox-LDL. (**D**) Indicated proteins in THP-1 cells were detected by IB assay. (**E**, **F**) Transwell migration and invasion assays were performed in THP-1 cells. Bar= 20μM. (**G**) MTT assay was used to analyze the viability of HUVEC cell after incubated with THP-1 cells. Data are representative of at least three independent experiments and are presented as mean ± SD. ns, not statistically significant; *, P < 0.05. **, P < 0.01.

To further examine the function of *PTPN2* in macrophages, we used several concentrations of XIX, a *PTPN2* inhibitor, to treat with THP-1 cells. The expression of *PTPN2* and the secretion of IL-12 and IL-1β were detected. Results showed that XIX with 0.25uM, 0.5uM, and 1.0uM could inhibit the expression of PTPN2 protein, and the effect of inhibition was more obvious when the concentration of XIX increased (Figure 4A). Furthermore, we combined XIX (1.0 μM) and ox-LDL to treat with THP-1 cells and examined the secretion of IL-12 and IL-1β. Compared with the group which only treated with ox-LDL, the mRNA of *IL-12* and *IL-1β* were increased in the XIX + ox-LDL group (Figure 4B). Consistently, the secretion of IL-12 and IL-1β were also enhanced in the XIX + ox-LDL group (Figure 4C). In addition, we detected the phosphorylation levels of p65, p38, and STAT3 proteins in different groups. Results showed that the levels of p-p65, p-p38, and p-STAT3 proteins were higher in XIX + ox-LDL group than in ox-LDL group (Figure 4D). We also examined the effect of XIX on the migration, invasion, and proliferation ability of THP-1 cells. Compared with the group which treated with ox-LDL alone, the metastatic and invasion capacity of cells in the XIX + ox-LDL group were enhanced (Figure 4E, 4F). MTT assay showed that the proliferation ability of cells was enhanced in the XIX + ox-LDL group (Figure 4G). These results showed that, similar to the shRNA of *PTPN2*, XIX combined with ox-LDL could also induce the phosphorylation of p65, p38 and STAT3, enhance the secretion of IL-12 and IL-1β, and promote cell proliferation, invasion and metastasis.

### *PTPN2* can be a potential treatment target of atherosclerosis

**Figure 5:**
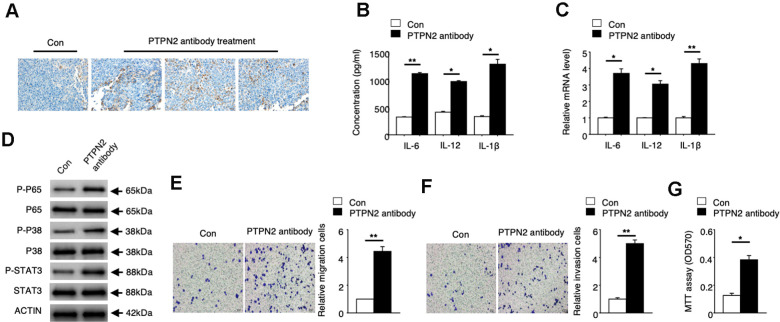
***PTPN2* can be a potential atherosis treatment target.** PBS control and PTPN2 antibody were injected into ApoE^-/-^ mice by tail vain injected. (**A**) Immunohistochemistry of CD68 in aortic roots of ApoE^-/-^ mice which treated with PBS or PTPN2 antibody were performed. (**B**) ELISA assay was used to analyze the production of IL-6, IL-12 and IL-1β in macrophages. (**C**) The mRNA levels of *IL-6, IL-12* and *IL-1β* in macrophages were analyzed by qRT-PCR assay. (**D**) IB assay was used to detect the expression of indicated proteins in macrophages. (**E**, **F**) Transwell migration and invasion assays in macrophages were performed. Bar= 20μM. (**G**) MTT assay was used to analyze the viability of HUVEC cell which incubated with macrophages. Data are representative of at least three independent experiments and are presented as mean ± SD. ns, not statistically significant; *, P < 0.05. **, P < 0.01.

To explore the potential application value of *PTPN2* on the treatment of atherosclerosis, we performed an in vivo assay in APOE^-/-^ mice. PBS control or PTPN2 antibody were injected into APOE^-/-^ mice by tail vein injection. Expression of PTPN2 proteins were detected by immunohistochemistry assay. The mRNA and protein levels of IL-6, IL-12 and IL-1β were analyzed by qRT-PCR assay and ELISA assay. Immunohistochemistry assay showed that PTPN2 antibody could reduce the expression of PTPN2 in macrophages of mice (Figure 5A). The mRNA and protein levels of IL-6, IL-12 and IL-1β was increased in macrophages when treated with PTPN2 antibody (Figure 5B, 5C). The phosphorylation levels of p65, p38, and STAT3 protein were also elevated after treatment with PTPN2 antibody (Figure 5D). Further research revealed that the ability of metastasis and invasion were enhanced when treatment with PTPN2 antibody (Figure 5E, 5F). MTT experiments showed that PTPN2 antibody could significantly promote the proliferation of macrophages (Figure 5G). These results indicated that *PTPN2* plays a negative role in the occurrence of atherosclerosis by inhibiting the secretion of inflammatory cytokines, and may be a potential treatment target of atherosclerosis.

## DISCUSSION

PTPN2 is an enzyme for protein de-phosphorylation, and cloned from T cell cDNA library [30]. It is abnormally high expression in lymphocytes and functions as a negative regulator in multiple inflammatory signaling pathways [31–33]. Abnormal expression of *PTPN2* will lead to the occurrence of many inflammatory diseases [25, 26]. In vivo experiments have shown that deletion of *PTPN2* will lead to severe systemic inflammation, multiple organ dysfunction, and die within 4-5 weeks [34, 35]. All of these researches indicate that *PTPN2* is closely related to the occurrence of inflammation. One typical characteristic of atherosclerosis is the systemic inflammation. Therefore, we analyzed the expression of *PTPN2* in APOE^-/-^ mice model of atherosclerosis. As we expected, the mRNA and protein expression levels of PTPN2 were significantly decreased in inflammatory mice. Deletion of *PTPN2* could induce the secretion of inflammatory cytokines and promote the proliferation, invasion and metastasis of macrophages. These works suggested that *PTPN2* plays an anti-inflammatory role in the occurrence of atherosclerosis, and the absence of *PTPN2* may lead to the persistent inflammation in macrophages.

Macrophage can be divided into M1 and M2 phenotype. M1 phenotype is a type of pro-inflammatory macrophages which can secrete inflammatory factors [36]. This will lead to the recruitment of inflammatory cells, and cause abnormal events such as thrombosis [37]. Conversely, M2 phenotype of macrophage is an anti-inflammatory cell. It will block the recruitment of inflammatory cells, inhibit the release of inflammatory factors, reduce the formation of foam cells, and relieve the development of atherosclerosis [38, 39]. To explore the differentiation process of monocyte toward M1 phenotype is very important for atherosclerosis treatment. In this study, we investigated that whether *PTPN2* can induce monocytes toward M1 phenotype. The results showed that *PTPN2* deficiency could significantly enhance the THP-1 cells and U937 cells toward M1 phenotype but not toward M2 phenotype. The proportion of F4/80^+^ CD11C^+^ positive cells was increased after deletion of *PTPN2*. The levels of IL-6, TNF-a and iNOS, which are associated with M1 polarization, were also notably increased. These results showed that *PTPN2* deficiency can not only regulate the proliferation, invasion and metastasis, but also induce monocyte toward M1 phenotype of macrophages.

p65 is a transcription factor that encoded by *RELA* gene and is also known as the nuclear factor NF-κB p65 subunit [40, 41]. It is a key subunit of NF-κB and involves in the formation of heterodimers, nuclear translocations, and activation of NF-κB signal pathway [42]. p38 is a protein associated with MAPK signaling pathway. It can respond to stress stimuli and participate in regulating various biological processes, such as cell differentiation, apoptosis, and autophagy [43, 44]. STAT3 is the transcriptional activator of JAK-STAT signaling pathway and activated by the phosphorylation of receptor-associated Janus kinase (JAK) [45, 46]. The JAK-STAT signaling pathway can regulate the release of multiple inflammatory factors in various diseases [47]. In this study, we found that deletion of *PTPN2* could promote the phosphorylation of p65, p38 and STAT3, and activate the release of related inflammatory factors. We also performed the similar experiment in mice by PTPN2 antibodies. Results showed that loss of *PTPN2* enhanced the phosphorylation of p65, p38 and STAT3, and then activated the related signaling pathways. These results indicate that *PTPN2* may play a negative role in the occurrence of macrophage inflammation by regulating the dephosphorylation of p65, p38 and STAT3.

In summary, we have preliminary studied the molecular mechanism of atherosclerosis by constructing an APOE^-/-^ mice model. We found that the expression of *PTPN2* is negative correlation with inflammation in APOE^-/-^ mice. shRNA of *PTPN2* could significantly enhance the secretion of inflammatory cytokines in macrophages, induce monocyte toward M1 phenotype of macrophages, and promote cell proliferation, invasion and metastasis. *PTPN2* inhibitor XIX combined with ox-LDL could observed a similar phenomenon as the absence of *PTPN2*. In vivo experiments revealed that PTPN2 antibody could reduce the protein level of PTPN2, promote the secretion of inflammatory cytokines, and induce the proliferation, invasion and metastasis of macrophages. In a word, in vitro and in vivo results suggested that *PTPN2* has a negative role on the regulation of macrophage inflammation in atherosclerosis, and it may become a potential target for atherosclerosis treatment.

## MATERIALS AND METHODS

### Atherosclerosis model in mice

All of animal assays in this study were carried out and approved by Animal Care Committee of Jiangxi Provincial People's Hospital Affiliated to Nanchang University according to the guidelines. ApoE^-/-^ mice (6-week old male) were fed with high-fat diet (HFD) and induced to be atherosclerosis mice model. 12 weeks later, all ApoE^-/-^ mice were killed by cervical dislocation, and the aortic roots were isolated and frozen in liquid nitrogen for subsequent experiments. PTPN2 antibody were intravenously injected through tail vein of ApoE^-/-^ mice. Macrophages in aortic roots were isolated by use of tissue lymphocyte separation solution (TBD, Tianjin, China) according to the protocol, and cultured in DMEM medium (Invitrogen, USA) with 1% penicillin/streptomycin (Sigma-Aldrich, USA), used in following experiments.

### Cell culture

THP-1 cells were purchased from the ATCC (Manassas, USA). RPMI1640 culture medium (Invitrogen, USA) was used for the culture of this cells. 10% heat-inactivated FBS (Invitrogen, USA) and 1% penicillin/streptomycin (Sigma, USA) were added in the medium. These cells were incubated in a humidified atmosphere of 5% CO2 at 37° C. PTP inhibitor XIX is purchased from Sigma Aldrich. The Lot No. of PTP inhibitor XIX is 540215.

### Enzyme-linked immunosorbent assay (ELISA)

The protein expression of IL-6, IL-12 and IL-1β were detected by ELISA kits (R&D Systems, Minneapolis, MN, USA) following the manufacturer's protocols.

### RNA isolation and qRT-PCR assay

Total RNA was extracted using the RNAiso Plus and reversely transcribed into cDNA using the Prime Script RT Master Mix. SYBR Premix Ex Taq II (Takara Bio, China) was used for qPCR assay. The primers used in human cells are as follows:

PTPN2-Forward (5'-3'): CATGCTGAACCGCATTGTGGAG,

PTPN2-Reverse (5'-3'): GACAAGAGCTTCACACTGAATCC;

IL-6-Forward (5'-3'): AGACAGCCACTCACCTCTTCAG,

IL-6-Reverse (5'-3'): TTCTGCCAGTGCCTCTTTGCTG;

IL-12-Forward (5'-3'): TGCCTTCACCACTCCCAAAACC,

IL-12-Reverse (5'-3'): CAATCTCTTCAGAAGTGCAAGGG;

IL-1β-Forward (5'-3'): CCACAGACCTTCCAGGAGAATG,

IL-1β-Reverse (5'-3'): GTGCAGTTCAGTGATCGTACAGG;

iNOS-Forward (5'-3'): GCTCTACACCTCCAATGTGACC,

iNOS-Reverse (5'-3'): CTGCCGAGATTTGAGCCTCATG;

TNF-α-Forward (5'-3'): CTCTTCTGCCTGCTGCACTTTG,

TNF-α-Reverse (5'-3'): ATGGGCTACAGGCTTGTCACTC;

Arginase-1-Forward (5'-3'): TCATCTGGGTGGATGCTCACAC,

Arginase-1-Reverse (5'-3'): GAGAATCCTGGCACATCGGGAA;

Ym-1-Forward (5'-3'): TACTCACTTCCACAGGAGCAGG,

Ym-1- Reverse (5'-3'): CTCCAGTGTAGCCATCCTTAGG;

IL-10-Forward (5'-3'): TCTCCGAGATGCCTTCAGCAGA,

IL-10-Reverse (5'-3'): TCAGACAAGGCTTGGCAACCCA;

GAPDH-Forward (5'-3'): GTCTCCTCTGACTTCAACAGCG,

GAPDH-Reverse (5'-3'): ACCACCCTGTTGCTGTAGCCAA.

The primers used in mouse cells are as follows:

IL-12-Forward (5'-3'): ACGAGAGTTGCCTGGCTACTAG,

IL-12-Reverse (5'-3'): CCTCATAGATGCTACCAAGGCAC;

IL-6-Forward (5'-3'): TACCACTTCACAAGTCGGAGGC,

IL-6-Reverse (5'-3'): CTGCAAGTGCATCATCGTTGTTC;

IL-1β-Forward (5'-3'): TGGACCTTCCAGGATGAGGACA,

IL-1β-Reverse (5'-3'): GTTCATCTCGGAGCCTGTAGTG;

GAPDH-Forward (5'-3'): CATCACTGCCACCCAGAAGACTG,

GAPDH-Reverse (5'-3'): ATGCCAGTGAGCTTCCCGTTCAG.

The Applied Biosystems 7900 Real Time PCR System (Applied Biosystems, USA) was used according to the manufacturer's instruction. CT values were recorded and expression of genes was calculated using the equation 2^-ΔΔCt^.

### Transfection of shRNA

Scrambled shRNA control and PTPN2-shRNA were transfected into THP-1 cells using Lipofectamine 2000 (Thermo Fisher) in 24-well plates. THP-1 cells were incubated for 18 h with in Opti-MEM containing 3μL Lipofectamine 2000 per 2×105 cells and 20-50 nM shRNA according to the instructions. The sequence of shRNA are as follows:

shRNA-1-F: GTGTGAAGCTCTTATCTGA,

shRNA-1-R: TCAGATAAGAG CTTCACAC;

shRNA-2-F: GCTCTTATCTGAAGATGTA,

shRNA-2-R: TACATCTTCAGATAAGAGC.

### Western blot analysis

Total proteins were extracted with SDS lysis buffer, and then separated by 10% SDS polyacrylamide gels. Proteins were transferred to the PVDF membrane after electrophoresis and blocked with 5% defatted milk for 1 hour. The blots were incubated with primary antibodies overnight, and then incubated with goat-anti-mouse IgG or goat-anti-rabbit IgG. β-actin or GAPDH was used as a loading control. The primary antibodies: anti-p38(1:1000, Abcam), anti-p-p38 (1:1000, Cell Signaling Technology), anti-p65 (1:1000, Cell Signaling Technology), anti-p-p65 (1:1000, Cell Signaling Technology), anti-STAT3 (1:1000, Cell Signaling Technology), anti-p-STAT3 (1:1000, Abcam), anti-PTPN2 (1:1000, Cell Signaling Technology), and anti-β-actin (1:5000, Cell Signaling Technology) were used in this study.

### Confocal microscopy assay

Macrophages were isolated from the aortic roots of mice and were incubated with primary antibodies of PTPN2 and CD11b (CST, USA) and following secondary fluorescence-labelled antibodies (CST, USA). DAPI solution was applied to stain cell nucleus. Cells were scanned under the confocal laser scanning microscope.

### EDU assay

EDU assay (Beyotime, Nanjing, China) was carried out to determine macrophages proliferation. 1x10^4^ cells were incubated in a 96-well plate overnight and then transfected by shRNA-1 or shRNA-2. The cell viability was detected after adding EDU solution to each well and incubating. Immunofluorescence analysis was performed for EDU detection.

### Cell proliferation, migration and invasion assay

MTT assay was performed to analyze the proliferation ability of cells. 2×10^5^ cells were plated in 24-well plates and cultured for 3 days. Cell viability was evaluated by the absorbance of 490nm (OD490) according to the instructions. 500 cells were plated in 6-well plates and cultured for 10-14 days in colony formation assay. Cell viability was evaluated by the count of colonies. 2×10^5^ cells were seeded into the chamber (migration) or matrigel coated chambers (invasion) and incubated 24h or 36h. The migration and invasion cells were evaluated by the count of five randomly selected optical fields.

### Flow cytometry assay

The THP-1 cells which transfected with shRNA-1 or shRNA-2 of PTPN were harvested, and the Annexin V-APC kit (eBioscience, USA) was performed to evaluate the apoptosis of THP-1 cells. Antibodies against F4/80, CD11b, CD11c, CD206 and controls and secondary antibodies (eBioscience, USA) were incubated with cells according to the manufacturers’ instructions. All experiments were performed independently in triplicate.

### Statistical analysis

Student's t-test was carried out to analyze the difference between groups, and it is statistical significance when P value was less than 0.05. At least three independent experiments were performed for data analysis. GraphPad Prism (Version5.0) were used for the statistical analysis.
